# Cases of Reflex and Infantile Paralysis

**Published:** 1862-07

**Authors:** 


### CASES OF REFLEX AND INFANTILE PARALYSIS.

(*Under the care of Dr. Brown-Sequard.*)

In closely contrasting the cases of paralysis under the care of Dr. Brown-Sequard, it is observable that in all the great number of cases which come under treatment by that physician, he rarely fails to detect indications of paralysis of both sides of the body. It is very common to read, to hear of, and even to see, cases of so-called paralysis of one arm or of one leg, or solely of one side of the body; but the accurate tests which Dr. Brown-Sequard applies almost invariably bring out proofs that parts of the body on the other side also participate. The universality of this observation has an interesting bearing upon the physiology of the brain, since it seems to point to the fact that there is no complete decussation of the brain-fibres, but that some remain to be concerned in the innervation of the same side of the body. In the treatment of paralysis, Dr. Brown-Sequard first ascertains by the proper inquiries whether the disease was originated in the membranes or the substance of the nervous centres, and then proceeds to limit the diagnosis to the precise point and special character of the lesion. Iodide of potassium is a remedy largely and usefully employed here. Local blistering, the ferro-citrate of strychnine, together with blood and nervine tonics are the other most common remedies. Amongst the most interesting are the causes of reflex paralysis, of which the study opens up comparatively a new field of observation, and suggests therapeutical resources fertile and good.

A remarkable case seen last week was that of a child who was stated to have "caught cold" eighteen weeks before, and who was the subject of a reflex paralysis of the eyelids and of some of the muscles of the eye. There was ptosis, prominence of the ball, and incapacity of abducting or adducting the eye, or of executing its rolling movements. Here, then, there was paralysis of the third, fourth, sixth, and seventh nerves. It was certainly reflex, for the iris was contracted firmly, so that one branch of the third was irritated and not paralysed. Any central cause which must have operated by causing pressure both behind and in front of the pons Varolii must, indeed, have been of considerable magnitude. But the cause was evidently reflex. The treatment advised was iron with minute doses of strychnine, and to be carefully watched; local Faradization, or the local application of heat by cloths to the surface.

In such a case, accurate diagnosis is the main step to cure. In investigating the cases of paralysis amongst children, Dr. Brown-Sequard points out occasionally the evils which have accrued from the application of term infantile paralysis to the paralytic affections of children, as though they were of one generic kind, and to be treated on a uniform general principle, and apart from the varied forms of paralysis seen in older persons. Dr. Brown-Sequard also sometimes points out cases of paralysis arising from a very wide range of differing causes almost as great as that in the case of adults. In one child it has followed an eruptive fever, and is the result of a sudden effusion into the ventricles of the brain; in another, the child is observed to cry out at night, to start in his dreams, and to raise his hand to his head. Here tubercular meningitis may be looked for. In a third, peripheral irritation may be discovered. These distinctions are of the utmost importance in treatment; and, before any course is undertaken, it is necessary to make out the cause and character of the paralytic affection.—*London Lancet.*

